# Study on Evaluation Model of Emergency Rescue Capability of Chemical Accidents Based on PCA-BP

**DOI:** 10.1155/2021/8869608

**Published:** 2021-01-13

**Authors:** Jianghong Liu, Junfeng Wu, Weisi Liu

**Affiliations:** College of Ocean Science and Engineering, Shanghai Maritime University, Shanghai 201306, China

## Abstract

The emergency management of chemical accidents plays an important role in preventing the expansion of chemical accidents. In recent years, the evaluation and research of emergency management of chemical accidents has attracted the attention of many scholars. However, as an important part of emergency management, the professional rescue team of chemicals has few evaluation models for their capabilities. In this study, an emergency rescue capability assessment model based on the PCA-BP neural network is proposed. Firstly, the construction status of 11 emergency rescue teams for chemical accidents in Shanghai is analyzed, and an index system for evaluating the capabilities of emergency rescue teams for chemicals is established. Secondly, the principal component analysis (PCA) is used to perform dimension reduction and indicators' weight acquisition on the original index system to achieve an effective evaluation of the capabilities of 11 rescue teams. Finally, the indicators after dimensionality reduction are used as the input neurons of the backpropagation (BP) neural network, the characteristic data of eight rescue teams are used as the training set, and the comprehensive scores of three rescue teams are used for verifying the generalization ability of the evaluation model. The result shows that the proposed evaluation model based on the PCA-BP neural network can effectively evaluate the rescue capability of the emergency rescue teams for chemical accidents and provide a new idea for emergency rescue capability assessment.

## 1. Introduction

Due to the properties of hazardous chemicals, such as toxicity, corrosiveness, explosiveness, flammability, and combustion support, there are huge risks in their production, transportation, storage, sales, use, and disposal. Once a hazardous chemical accident occurs, it is easy to cause many casualties, huge property losses, and serious environmental pollution and bring catastrophic consequences to both enterprises and the society. For example, the explosion of a dangerous good warehouse in Tianjin Port on August 15, 2015, resulted in 165 deaths, 798 casualties, and 8 missing. The direct economic loss reached 6.866 billion yuan [1]. Therefore, the emergency treatment of chemical accidents must be timely and efficient to prevent accidents from expanding and causing even greater losses.

In recent years, the assessment of emergency management capabilities for hazardous chemical accidents has attracted the attention of many scholars. Wang et al. [2] proposed a disaster management control capability assessment model based on the Capability Maturity Model (CMM). This model evaluates the capability of the organization from eight aspects and divides the capability assessment results into four levels, which provides general assessment guidelines for different types of emergency management organizations. Lin [3] analyzed the nature of emergency rescue capabilities from the perspective of the entire city and established an urban emergency rescue capability evaluation system based on AHP and Fuzzy Comprehensive Evaluation (FCE). Yang et al. [4] analyzed many factors that affect the emergency capacity of enterprises, established an emergency capacity evaluation index system, and determined the weight for each indicator through the Analytic Hierarchy Process (AHP). Yu and Guan [5] analyzed the current situation and difficulties of emergency treatment of hazardous chemical accidents, discussed the emergency training system, and provided a reference for improving the emergency capabilities of professional teams for fire and hazardous chemical rescue. Zhu et al. [6] used Bayesian networks to propose a framework for dynamically evaluating explosion accidents in chemical plants to support prevention, management, and real-time warning. He et al. [7] established a Petri net model of emergency process of chemical accidents in order to evaluate the emergency capability, which can dynamically evaluate the emergency capability of chemical accidents.

In addition to the abovementioned traditional evaluation methods, the application of artificial neural networks to the evaluation of chemical accidents has made some progress. Yuan et al. [8] used back propagation neural networks, generalized regression neural networks and radial basis function neural networks to evaluate the safety production management of chemical companies and found that the prediction ability of the radial basis function neural network is more accurate. Aiming at the shortcomings of the current chemical production safety evaluation system and combining the knowledge of artificial neural networks, Yang established a new evaluation index system and proposed the advantages of applying neural networks to the chemical production safety evaluation system [9].

These studies mainly focus on the establishment of an emergency management assessment system for hazardous chemical accidents and the application of emergency assessment methods. Or, consider the emergency management activity itself as a project management process and study the capacity assessment model for emergency management control. However, as an important part of emergency management, the professional rescue team of hazardous chemicals has few evaluation models for their capabilities. In addition, traditional evaluation methods, such as Analytic Hierarchy Process (AHP), are greatly affected by human factors in the implementation process, and it is difficult to obtain objective evaluation results. When there are many evaluation indicators, it will complicate the structure of the artificial neural network model and increase the computational complexity. Shanghai is an important petrochemical and fine chemical industry base in China, with a solid chemical industry foundation. Through the assessment of the emergency response capabilities of the 11 professional rescue teams for hazardous chemicals in Shanghai, the capabilities of the rescue teams can be strengthened using targeted countermeasures.

In order to reasonably evaluate the capabilities of professional emergency rescue teams for hazardous chemical accidents, this study surveyed 11 professional rescue teams in Shanghai, analyzed the status of these teams, constructed a rescue capacity assessment index system, and built a rescue capability evaluation model combined with BP neural network. At the same time, in order to determine the indicators' weight and reduce the number of neurons in the input layer of the backpropagation (BP) neural network, the principal component analysis (PCA) was used to reduce the dimension of the evaluation index system and obtain weight. The dimensionality-reduced feature factors were used as the input units of the BP neural network. This method can not only reduce the influence of human factors in the evaluation process but also simplify the structure of the artificial neural network and reduce the computational complexity of the evaluation model. The trained BP neural network evaluation model can well evaluate the capabilities of professional emergency rescue teams for hazardous chemical accidents, providing a new idea for emergency rescue capability assessment.

## 2. Methods

### 2.1. Construction of Rescue Capability Evaluation Index System

Shanghai has a total of 11 emergency rescue teams for production safety, as shown in Table 1. At present, the 11 emergency rescue teams for production safety are managed by the company where they work. The Shanghai Emergency Management Bureau is responsible for providing business guidance. As a result of a thorough investigation, following construction problems were found with these teams:

1. Inefficient cooperation mechanism: there is a lack of coordination between the emergency rescue team and other departments. The team's responsibilities are unclear, and there is no unified command.
2. Slow emergency response: the lack of classification and corresponding response plans based on the type and scale of hazardous chemical accidents makes the emergency rescue scene more chaotic.
3. Inappropriate team building: the positioning of these rescue teams is unclear, and the rescue areas are not divided. There are no long-term full-time members in these rescue teams, and these team members have not received any specialized training in dealing with hazardous chemical accidents.
4. Insufficient emergency equipment, materials, and maintenance funds. The maintenance of professional rescue equipment for hazardous chemical accidents lacks government financial support. The necessary equipment and materials cannot be timely supplemented.
5. Noncompliant emergency handling. There is a dearth of norms and standards on the emergency handling of hazardous chemical accidents. There is no targeted emergency response plan for different hazardous chemical accidents. Due to the problem of team building, rescuers with nonprofessional characteristics, and temporary combination, it is difficult to conduct emergency response scientifically and quickly.

According to the above construction status, an emergency rescue team assessment index system was established. The emergency response capabilities of professional rescue teams for hazardous chemical accidents include the following elements:

1. Emergency cooperation: the main consideration is whether the division of responsibilities within the rescue team is reasonable and clear and whether smooth information can be communicated between various departments; whether the management of human resources has considered a perfect reward and punishment system, employee benefits and incentives; and whether the communication is efficient enough to ensure the normal operation of the emergency mechanism.
2. Emergency command: the emergency rescue of hazardous chemical accidents mainly includes two aspects: an emergency disposal plan and the emergency professional and technical personnel. Different schemes are needed to respond to different chemical accidents as the quantity and types of hazardous chemicals always vary between different plants and regions. Therefore, certain requirements are put forward for the pertinence and completeness of the emergency response plan and the allocation of emergency professional and technical personnel.
3. Emergency foundation: personnel, materials, equipment, and funds are the basis for emergency rescue of hazardous chemical accidents. In this study, the factors that affect basic emergency support are divided into four parts: (1) the emergency team, considering whether the stability, quantity, and quality of emergency personnel; (2) emergency equipment (including personnel protection equipment), the functionality, safety, quality, and quantity of equipment should satisfy the emergency disposal requirements; (3) whether emergency materials could meet different types of hazardous chemical accidents; (4), emergency funding, whether governments and enterprises had been given economic support to ensure better operation of the emergency rescue teams.
4. Training and education: consider the training of professional emergency rescue knowledge and skills. Assess the improvement of the emergency rescue ability of the corresponding emergency personnel.
5. Emergency drills: consider the workload, such as whether the number and time-frequency of drills is reasonable to meet the demands. In addition, the factors that need to be considered are the effects of the emergency drills, whether the personnel is familiar with the emergency procedures and more effective in responding to special chemical accidents through emergency drills.

Accordingly, this research proposed a rescue capability evaluation index system that includes 5 first-level indicators, 14 second-level indicators, and 28 third-level indicators, as shown in Table 2:

### 2.2. Dimension Reduction of Evaluation Indicators and Weight Acquisition

The above index system is too complicated; using the original indicator as the input unit of the BP neural network will face problems of such as high data dimensions, poor fitting effects, and inaccurate prediction results. Therefore, the principal component analysis (PCA) was required to reduce the dimensions of the indicators to eliminate the correlation [10].

Principal component analysis (PCA) is an important statistical method that uses the idea of dimensionality reduction to transform multiple indicators into a few comprehensive indicators. These comprehensive indicators are not explanatory but retain most of the original information [11]. The new comprehensive indicator is a linear combination of all the original indicators which remain independent of each other. The principal component analysis (PCA) can reduce the number of evaluation indicators, thereby reducing the number of neurons in the input layer to simplify the structure of the BP neural network.

In a geometric sense, the principal component analysis (PCA) method is to project the original data onto a new coordinate axis, which is the principal component. In order to enable the principal component to contain more information about the original data, the variance of the principal component must be maximized. The process of principal component analysis (PCA) is to find linear combination coefficients. The coefficients must maximize the variance of the principal components, and the sum of the squares of the coefficients must be equal to one. In addition, starting from the second principal component, each principal component must be independent of the existing principal components.

The specific steps of principal component analysis (PCA) are as follows:

Step 1: standardize the raw data.
Assuming the original data is an *n* × *m* matrix:
  (1) $$\begin{array}{c} X=\left[\begin{array}{cccc} x_{11} & x_{12} & \cdots & x_{1m}\\ x_{21} & x_{22} & \cdots & x_{2m}\\ \vdots & \vdots & \vdots & \vdots \\ x_{n1} & x_{n2} & \cdots & x_{nm} \end{array}\right]. \end{array}$$
The rows in the data matrix represent different samples, and the columns represent different evaluation indicators. It can be seen that there are *n* samples and *m* evaluation indicators. The matrix can be normalized by the following formula:
  (2) $$\begin{array}{c} x_{ij}^{*}=\frac{x_{ij}-\bar{x_{j}}}{\sqrt{\mathrm{var}\left(x_{j}\right)}},\;i=1,2,\ldots ,n;\;j=1,2,\ldots ,m, \end{array}$$
among them,
  (3) $$\begin{array}{c} \bar{x_{j}}=\frac{1}{n}\sum_{i=1}^{n}x_{ij}. \end{array}$$
Variance:
  (4) $$\begin{array}{c} \mathrm{var}\left(x_{j}\right)=\frac{1}{n-1}\sum_{i=1}^{n}{\left(x_{ij}-\bar{x_{j}}\right)}^{2},\;j=1,2,\ldots ,m. \end{array}$$
Step 2: calculate the correlation coefficient matrix of the sample indicators:
  (5) $$\begin{array}{c} R=\left[\begin{array}{cccc} r_{11} & r_{12} & \cdots & r_{1m}\\ r_{21} & r_{22} & \cdots & r_{2m}\\ \vdots & \vdots & \vdots & \vdots \\ r_{m1} & r_{m2} & \cdots & r_{mm} \end{array}\right], \end{array}$$
among them,
  (6) $$\begin{array}{c} r_{ij}=\frac{\mathrm{cov}\left(x_{i},x_{j}\right)}{\sqrt{\mathrm{var}\left(x_{i}\right)}\sqrt{\mathrm{var}\left(x_{j}\right)}}=\frac{\sum_{k=1}^{n}\left[\left(x_{ki}-\bar{x_{i}}\right)\left(x_{kj}-\bar{x_{j}}\right)\right]}{\sqrt{\sum_{k=1}^{n}{\left(x_{ki}-\bar{x_{i}}\right)}^{2}}\sqrt{\sum_{k=1}^{n}{\left(x_{kj}-\bar{x_{j}}\right)}^{2}}},\;n>1. \end{array}$$
Step 3: calculate the eigenvalue *λ*_*i*_(*i*=1,2,…, *m*) and eigenvector *v*_*i*_=(*v*_*i*1_, *v*_*i*2_, *v*_*i*3_,…, *v*_*im*_),  *i*=1,2,…, *m* of the correlation coefficient matrix.
Step 4: select the principal component *P*_*t*_.
All the eigenvalues *λ*_*i*_(*i*=1,2,…, *m*) are arranged in descending order. The larger the eigenvalue, the more the system information contained in the principal component. Calculate the contribution rate of each principal component by the following formula:
  (7) $$\begin{array}{c} \omega_{i}=\frac{\lambda_{i}}{\sum_{i=1}^{m}\lambda_{i}},\;i=1,2,\ldots ,m. \end{array}$$
It is generally considered that the cumulative contribution rate of the first *q* principal components exceeds 85% is reasonable, indicating that the total amount of system information they contain exceeds 85%. At this time, the principal component *P*_*t*_(*t*=1,2,…, *q*) is the characteristic index after dimensionality reduction.
Step 5: find the unit orthogonal feature vector *v*_*t*_=(*v*_*t*1_, *v*_*t*2_, *v*_*t*3_,…, *v*_*tm*_), *t*=1,2,…, *q* of the first *q* feature vectors. Each principal component is a linear combination of all the original indicators, and the coefficient *a*_*tj*_ is the element of the unit orthogonal eigenvector:
  (8) $$\begin{array}{c} a_{tj}=v_{tj},\;t=1,2,\ldots ,q;\;j=1,2,\ldots ,m. \end{array}$$
Then, the expression of the principal component *P*_*t*_ of the *n*-th sample is
  (9) $$\begin{array}{c} P_{1}=a_{11}x_{n1}+a_{12}x_{n2}+a_{13}x_{n3}+\cdots +a_{1m}x_{nm}\\ P_{2}=a_{21}x_{n1}+a_{22}x_{n2}+a_{23}x_{n3}+\cdots +a_{2m}x_{nm}\\ \vdots \\ P_{q}=a_{q1}x_{n1}+a_{q2}x_{n2}+a_{q3}x_{n3}+\cdots +a_{qm}x_{nm}. \end{array}$$
Step 6: the comprehensive evaluation function *F*_*n*_ of the *n*-th sample is shown in the following formula:

(10) $$\begin{array}{c} F_{n}=\omega_{1}P_{1}+\omega_{2}P_{2}+\cdots +\omega_{q}P_{q}. \end{array}$$

 The weight of the principal component is the contribution rate *ω*_*i*_, *i*=1,2,…, *q*.

### 2.3. Construction of Evaluation Model Based on BP Neural Network

**Figure 1:**
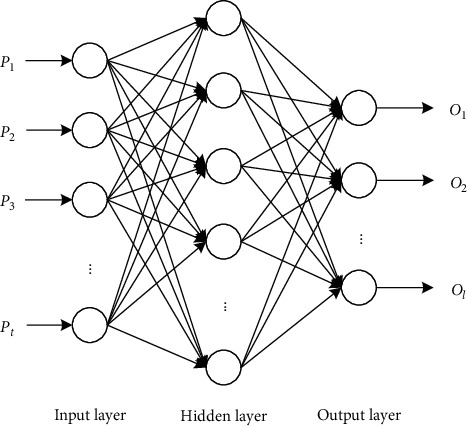
Structure of BP neural network.

BP neural network is a multilayer feedforward network model. Its network is mainly composed of three parts: input layer, hidden layer, and output layer. As shown in Figure 1, it can map *t*-dimensional data to *l*-dimensional data. The neurons in each layer of the BP neural network are not connected, and the output of the neurons in each layer only affects the output of the next layer. At the same time, the network will backpropagate errors during operation to continuously adjust the weights and thresholds of the network to achieve self-adjustment [12]. BP neural network is a nonlinear adaptive system, so it is more suitable for dealing with fuzzy or nonlinear problems. This method can effectively reduce subjective factors in the evaluation process and reduce the evaluation time [13].

The main parameter settings of the BP neural network are the number of network layers, the number of nodes in the hidden layer, the transfer function, and the training function, which will be introduced one by one as follows.

#### 2.3.1. Number of Network Layers

As the number of network layers increases, the structure of the BP neural network will become more and more complex. Correspondingly, the complex BP neural network will prolong the learning time and cause the phenomenon of “overfitting.” Through previous testing and research, the neural network is generally set up as a three-layer network, that is, the input layer, the hidden layer, and the output layer [14].

#### 2.3.2. Number of Hidden Layer Nodes

Too few hidden layer neurons may not train the desired network, or the trained network is not strong enough and has poor generalization ability. To contrary, too many hidden layer neurons will increase the learning time and the error may not be smaller. The number of neurons in the hidden layer can be obtained by the following empirical formula [15]:

(11) $$\begin{array}{c} b=\sqrt{t+o}+a, \end{array}$$

where *b* is the number of neurons in the hidden layer, *t* is the number of neurons in the input layer, *o* is the number of neurons in the output layer, and *a* is a constant between [1, 10].

#### 2.3.3. Transfer Function

There are three main transfer functions: logsig function, tansig function, and purelin function. The choice of the transfer function of the hidden layer and the output layer has a greater impact on the prediction accuracy of the BP neural network. The logsig transfer function is an S-type logarithmic function, the tansig function is an S-type hyperbolic tangent function, and both are nonlinear functions. After that, the purelin function is a linear function. Generally, the transfer function of the hidden layer selects the logsig function or the tansig function, and the transfer function of the output layer selects the purelin function. As far as the nonlinear transfer function is concerned, if the output of the samples is greater than zero, the logsig function is mostly used; otherwise, the tansig function is used.

#### 2.3.4. Training Function

The common training functions are as follows:

  trainlm: LevenbergMarquardt method
  traingd: gradient descent method
  traingdm: gradient descent method with momentum factor
  traingda: gradient descent method with adaptive learning rate
  traingdx: gradient descent method with adaptive learning rate and momentum factor

## 3. Data Processing

### 3.1. Data Sources

The abovementioned evaluation index system was determined by analyzing the construction status of 11 emergency rescue teams for hazardous chemical accidents in Shanghai. Then, this paper produced a score sheet for these professional rescue teams. To reduce the subjective arbitrariness when scoring, the results were divided into 5 levels, and each level was guaranteed to take a positive value, from the worst to the best, respectively, 1, 2, 3, 4, and 5 points. See Table 3 for scoring criterion. The scorer could select the corresponding score according to the actual situation.  Table 4 shows the scores of the 11 rescue teams:

### 3.2. Data Dimensionality Reduction and Weight Determination

In this research, SPSS 25.0 software was used to perform the principal component analysis (PCA) of the original data to achieve data reduction and weight determination. The eigenvalues of the correlation coefficient matrix between 28 indicators, the contribution rate and the cumulative contribution rate of the principal components (Table 5), and the factor load matrix (Table 6) could be automatically calculated through the factor analysis tool in SPSS software. As can be seen from Table 5, there are 7 eigenvalues greater than 1, and the cumulative contribution rate of the corresponding 7 principal components reaches 94.743%, which meets the requirement that the cumulative contribution rate is generally greater than 85%. However, as shown in Table 6, none of the last two of the 7 principal components exceeds 0.5, and the cumulative contribution rate of the first 5 principal components reaches 85.849%, which also meets the requirement of greater than 85%. Therefore, this study chose the first 5 principal components as input units of the final BP neural network. Although the unit orthogonal eigenvector could not be directly obtained by the factor analysis tool of SPSS software, it could be calculated by the relationship formula (12) between the unit orthogonal eigenvector and the factor load:

(12) $$\begin{array}{c} a_{tj}=\frac{L_{t}}{\sqrt{\lambda_{t}}},\;t=1,2,\ldots ,q;\;j=1,2,\ldots ,m, \end{array}$$

where *L*_*t*_ is the load of principal component *P*_*t*_. So the coefficients of the five principal component linear expressions were known (Table 7).

The contribution rates of the 5 principal components are weights, and the normalized weights are shown in Table 8. The specific values of the 5 principal components in the 11 rescue teams were obtained through Table 7 and formula (9), and the comprehensive scores of the emergency capabilities of the 11 rescue teams were finally calculated through formula (10), as shown in Table 9:

As can be seen from Table 9, among the 11 rescue teams, team 3 gets the highest score of 12.441 points, while team 8 gets the lowest score of only 7.541 points. The difference between the highest and lowest scores is 4.9 points. Compared with the original scoring Table 4, it can be found that team 3 scores 4 or 5 points except D23 and D24. The difference is that for team 8, except for D22, all other indicators are 1 or 3 points. Therefore, the comprehensive scores obtained by the principal component analysis method are consistent with actual situations and can effectively reflect the emergency response status of 11 rescue teams.

## 4. Implementation of BP Neural Network

### 4.1. Sample Data Normalization

Normalization refers to limiting the input and output data of the network to [0, 1] or [−1, 1] through the processing of variables, which can improve the efficiency of the transfer function and the accuracy of the output of the neural network. There is a maximum-minimum method to limit the data between [0, 1], and the function form is as follows:

(13) $$\begin{array}{c} x_{i}=\frac{\left(x_{i}-x_{\min}\right)}{\left(x_{\max}-x_{\min}\right)}. \end{array}$$

The formula that limits the data to [−1, 1] is

(14) $$\begin{array}{c} x_{i}=2\times \frac{\left(x_{i}-x_{\min}\right)}{\left(x_{\max}-x_{\min}\right)}-1. \end{array}$$

In this paper, the mapminmax function provided by MATLAB was used to obtain the normalized input and output data between [−1, 1]. The normalized data are shown in Table 10:

Teams 1 to 8 were used as training samples for the BP neural network, and teams 9 to 11 were used as prediction samples for the BP neural network.

### 4.2. Determination of the Transfer Function

In this study, the data were limited to [−1, 1] during normalization. It could be known from the above description of the transfer function that the tansig function should be used. Therefore, it was determined that the transfer function of the hidden layer was the tansig function, and the transfer function of the output layer was selected as the purelin function.

### 4.3. Selection of Training Function

In order to determine the fast and accurate training function, this research used BP neural network toolbox of MATLAB software to experiment the above five training functions and then compared the training results to choose. According to the previous principal component analysis (PCA), five input neurons have been identified, named *P*_1_, *P*_2_, *P*_3_, *P*_4_, and *P*_5_, and only one output neuron which was “comprehensive score.” From the above empirical formula, it could be known that the number of hidden layer neurons should be selected between [4, 13], and it was temporarily determined to be 9. The number of iterations and convergence accuracy were used as the evaluation indicators for the training function selection. Before using the above five training functions to perform prediction fitting on 8 training samples, the maximum number of iterations was set to 2000 and the target convergence accuracy was set to 0. The results are shown in Table 11:

From the above training results, it can be seen that the trainlm training function achieves high accuracy in only 5 steps. The trainlm training function has the fastest convergence speed, but it is easy to fall into a local minimum. The traingd function and trackingdm function converge slowly in practical applications, and the convergence accuracy is not as high as the other three training functions. The traingda function and traingdx function have greatly improved the convergence accuracy of training, but the traingdx function converges faster than the traingda function and the traingdx function can avoid falling into local minima due to the additional momentum term and adaptive learning rate. At the same time, the training precision of the traingdx function reaches 8.3968 × 10^−10^, which is consistent with the convergence accuracy in general cases. Therefore, the BP neural network model established in this paper used the traingdx function as the training function.

### 4.4. Setting of Training Parameters

In this study, two parameters of the BP neural network model were set. The maximum allowable error was set to 0.00001, the maximum number of learning times was set to 1000, and the remaining parameters adopted default values.

### 4.5. Determination of the Number of Hidden Layer Neurons

The value range of hidden layer neurons has been obtained through the empirical formula in the previous article. However, if the number of hidden layer neurons is too small, the ability of the neural network to obtain information from the sample is poor, and it is impossible to generalize and reflect the sample law. At the same time, if there are too many neurons, the irregular content in the sample may be learned and the phenomenon of “overfitting” may occur. Therefore, it is necessary to determine an optimal number of hidden layer neurons.

**Figure 2:**
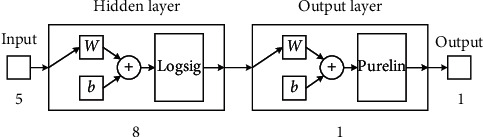
The structure of the BP neural network model.

The number of hidden layer neurons can be determined one by one [4, 13] through experiments. The number of training sessions was set to 1000, and the target accuracy was set to 0.00001. The number of iterations and the convergence accuracy of 10 trainings were compared to determine the appropriate number of hidden layer neurons. The training results are shown in Table 12:

As shown in Table 12, the convergence accuracy is on an order of magnitude, but there is some gap in the number of iterations. When the number of hidden layer neurons is 8, 9, 12, and 13, the number of iterations is small. From the perspective of simplifying the structure of the BP neural network, this study determined that the number of hidden layer neurons was 8. The resulting structure of BP neural network is shown in Figure 2:

### 4.6. Computational Complexity

The number of neurons in a neural network has an important impact on the computational complexity. When the number of neurons increases, the network calculation becomes more complex. The complexity of calculating the gradient of a certain layer is *O*(*D*^3^), assuming that the number of neurons in this layer is *D*. This study uses principal component analysis to reduce the number of neurons in the input layer of the neural network and obtained the final structure of neural network: 5 neurons in the input layer, 8 neurons in the hidden layer, and 1 neuron in the output layer. Therefore, the final calculation of the entire neural network is *O*(5^3^+8^3^+1^3^). In contrast, the computational complexity of the input layer of the neural network that directly uses all the evaluation indicators as the input layer neurons is *O*(28^3^), which is far greater than the computational complexity of the optimized neural network.

## 5. Simulation and Result Analysis of BP Neural Network Evaluation Model

### 5.1. Training of BP Neural Network

**Figure 3:**
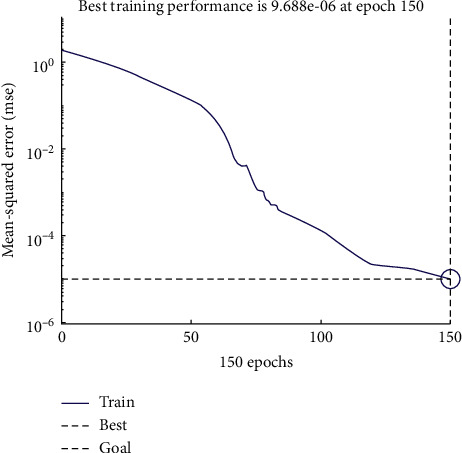
Mean square error trace.

After the above discussion, the parameters of the BP neural network evaluation model had been determined. The first 8 teams were now used as training samples to train the BP neural network. The training result is shown in Figure 3. After 150 iterations, the mean-squared error of the neural network reaches 9.688*e* − 6. As shown in Table 13, the maximum relative error between the training results and the comprehensive score is only 0.146%, indicating that the BP neural network has reached the training requirements.

### 5.2. Simulation of BP Neural Network

The trained BP neural network evaluation model was used to predict the comprehensive scores of the remaining three rescue teams. The relative errors between the predicted results and the theoretical values are shown in Table 14. It can be known from Table 14 that the maximum relative error is 6.658%, which indicates that the generalized ability of the trained BP neural network evaluation model can meet the emergency rescue capability assessment needs of rescue teams.

## 6. Conclusion

1. The capacity evaluation index system for the emergency rescue teams for hazardous chemical accidents proposed in this study was considered from five aspects: emergency cooperation, emergency command, emergency foundation, training and education, and emergency drills. A total of 5 first-level indicators, 14 second-level indicators, and 28 third-level evaluation indicators were proposed, which fully considered the factors affecting emergency rescue capabilities and were relatively comprehensive and objective.
2. The dimensionality reduction of the evaluation index was achieved by the principal component analysis (PCA) method, and the weights and comprehensive scores of 11 emergency rescue teams were obtained. According to the original index scoring table, it is found that the comprehensive scores obtained by the principal component analysis are in line with actual situations and can well reflect the emergency rescue capabilities of the 11 rescue teams.
3. After the dimensionality reduction by the principal component analysis (PCA) method, the number of neurons in the input layer of the BP neural network was greatly reduced and the structure of the BP neural network was simplified. At the same time, the computational complexity of the neural network evaluation model has also been reduced. Teams 1 to 8 were used as training samples for the BP neural network evaluation model, and teams 9 to 11 were used as test samples for verifying the model. The trained BP neural network evaluation model showed good generalization ability, and the highest relative error with the theoretical comprehensive score of the test sample was 6.658%, which could meet the needs of emergency rescue capability assessment.

The aim of this paper is to assess the capability of the emergency rescue team for hazardous chemical accidents in order to better understand the current situation of these rescue teams. This understanding will support the improvement the capability of these teams in a targeted way. In addition, competent authorities could use such assessments to improve their management level. The above research results showed that it is feasible to use the PCA-BP neural network-based evaluation model to evaluate the capability of emergency rescue teams for hazardous chemical accidents, which provides a new idea for emergency rescue capability assessment.

## Figures and Tables

**Table 1:** The 11 emergency rescue teams for hazardous chemicals in Shanghai.

Serial number	Team name
Team 1	Emergency Rescue Team of Shanghai Jiemeng Chemical Co., Ltd.
Team 2	Chemical Accident Emergency Rescue Center of Shanghai Institute of Occupational Disease for Chemical Industry
Team 3	Emergency Rescue Team of Testing Center of Shanghai Research Institute of Chemical Industry Co., Ltd.
Team 4	Emergency Rescue Team of Shanghai Huayi Energy Chemical Co., Ltd.
Team 5	Fire Brigade of Bayer
Team 6	Emergency Rescue Team of Shanghai Gaoqiao Petrochemical Chemical Transportation Co., Ltd.
Team 7	Chemical Rescue Team of BASF
Team 8	Rescue Team of Shanghai Zhongshi Chemical Logistics Co., Ltd.
Team 9	Emergency Rescue Team of Shanghai Chlor-Alkali Chemical Co., Ltd.
Team 10	Fire Brigade of Refining Department of Sinopec Shanghai Gaoqiao Petrochemical Co., Ltd.
Team 11	Ambulance Team of Jinshan Hospital of Fudan University

**Table 2:** Index system for evaluating the capabilities of emergency rescue teams for hazardous chemical accidents.

First-level indicators	Second-level indicators	Third-level indicators
*Emergency cooperation B1*	Duties within the team C1	Post setting D1
		Clarity of post responsibilities D2
		Duty fulfillment D3
	Information exchange between departments C2	Smooth communication D4
		Timeliness of information transfer D5
		Integrity of information transfer D6
	Human resources C3	Reward and punishment system D7
		Salary and benefits D8
		Career opportunities D9
	Communication network C4	Basic intercom equipment D10
		Advanced communication system and platform D11

*Emergency command B2*	Emergency plan C5	Whether the emergency response plan is scientific and accurate D12
		Whether the emergency response plan is targeted D13
		Whether the emergency treatment plan is operable D14
	Emergency expert C6	Whether emergency experts are qualified for emergency work D15

*Emergency foundation B3*	Emergency team C7	Whether there is sufficient staff D16
	Emergency equipment C8	Completeness of emergency rescue equipment D17
		Completeness of personal protective equipment D18
		The ability of emergency rescue equipment to control and reduce the impact of an accident D19
		Protective capabilities of personal protective equipment D20
	Emergency materials C9	Completeness of emergency materials D21
		Replenishment of emergency materials D22
	Emergency funding C10	Emergency funding support in Shanghai D23
		Territory emergency funding support D24

*Training and education B4*	Emergency rescue expertise training C11	Emergency rescue expertise training D25
	Emergency rescue professional skills training C12	Emergency rescue professional skills training D26

*Emergency drills B5*	Workload of emergency drill C13	Workload of emergency drill D27
	Effect of emergency drill C14	Effect of emergency drill D28

**Table 3:** The scoring criteria of the original evaluation index.

Evaluation index	Points	Scoring criteria
*Postsetting D1*	5	The emergency team has necessary positions, and the position setting is very reasonable, so that the team can operate most efficiently
	4	The emergency team has necessary positions, and the position setting is reasonable, so that the team can operate effectively
	3	The emergency team has necessary positions, and the position setting is not very reasonable, so that the team can operate
	2	The emergency team does not fully satisfy the necessary positions, but it does not affect the operation of the team
	1	The emergency team did not meet the necessary positions and affected the operation of the team

*Clarity of post responsibilities D2*	5	Very clear and no ambiguous parts
	4	Clear, but a little bit ambiguous
	3	More clear, but there are still some unclear parts
	2	Partly clear, but there are still many unclear parts
	1	Unclear

*Duty fulfillmentD3*	5	Personnel in all positions perform job duties
	4	The majority of positions perform their duties
	3	More than half of the staff perform job duties
	2	Personnel in some positions perform job duties
	1	A very small number of personnel perform job duties

*Smooth communication D4*	5	Very smooth, no need for improvement
	4	Smooth, best if it can be improved
	3	It is smoother and can be improved
	2	Generally smooth, needs improvement
	1	Not smooth

*Timeliness of information transfer D5*	5	Timely and effective
	4	Not very timely but still effective
	3	Timely but not very effective
	2	Timely but almost ineffective
	1	Not timely and ineffective

*Integrity of information transfer D6*	5	Both accurate and complete
	4	Accurate and relatively complete
	3	Accurate but not very complete
	2	Not very accurate but complete
	1	Inaccurate and incomplete

*Reward and punishment system D7*	5	Effectively mobilize the enthusiasm of the team members, and the effect is very good
	4	Aroused the enthusiasm of the team members, and the effect was good
	3	Aroused the enthusiasm of the team members, but the effect was average
	2	Aroused the enthusiasm of the team members, but the effect was very small
	1	Cannot mobilize the enthusiasm of team members

*Salary and benefits D8*	5	Very satisfied
	4	Satisfied
	3	Generally satisfied
	2	Not very satisfied
	1	Not satisfied

*Career opportunities D9*	5	Have career development prospects, can maintain the stability of the team members, and attract new team members to join
	4	Have career development prospects, can maintain the stability of the team members, but cannot attract new team members to join
	3	Have certain career development prospects and can basically maintain the stability of the team members
	2	There are very few career development prospects, and it is difficult to maintain the stability of the team members
	1	There is almost no career development prospects, and the stability of team members cannot be maintained

*Basic intercom equipment D10*	5	Very satisfied
	4	Satisfied
	3	Generally satisfied
	2	Not very satisfied
	1	Not satisfied

*Advanced communication system and platform D11*	5	Have
	1	Have not

*Whether the emergency response plan is scientific and accurate D12*	5	The emergency response plan is highly scientific and correct and can handle emergency work very effectively
	4	The emergency response plan has strong scientificity and accuracy and can handle emergency work
	3	The emergency response plan is generally scientific and correct; although it can handle emergency work, there are some problems
	2	The scientificity and accuracy of the emergency response plan are low, and there are problems and difficulties in handling emergency work
	1	The emergency response plan is hardly scientific and correct and cannot handle emergency work

*Whether the emergency response plan is targeted D13*	5	The emergency response plan has strong pertinence
	4	The emergency response plan has relatively strong pertinence
	3	The emergency response plan is generally targeted
	2	The emergency response plan is less targeted
	1	The emergency response plan is hardly targeted

*Whether the emergency treatment plan is operable D14*	5	The emergency response plan has strong operability
	4	The emergency response plan has relatively strong operability
	3	The operability of the emergency response plan is general
	2	The operability of the emergency response plan is weak
	1	The emergency response plan is almost inoperable

*Whether emergency experts are qualified for emergency work D15*	5	Totally satisfied
	4	Relatively satisfied
	3	Generally satisfied
	2	Not very satisfied
	1	Not satisfied

*Whether there is sufficient staff D16*	5	Totally satisfied
	4	Relatively satisfied
	3	Generally satisfied
	2	Not very satisfied
	1	Not satisfied

*Completeness of emergency rescue equipment D17*	5	Fully equipped and able to perform all emergency rescue activities
	4	The equipment is relatively complete and can perform most of the emergency rescue activities
	3	The equipment needs to be supplemented and perfected but can perform basic emergency rescue activities
	2	The equipment is not perfect, and there are certain difficulties in performing emergency rescue activities
	1	The equipment is not perfect, and there are difficulties in performing emergency rescue activities

*Completeness of personal protective equipment D18*	5	The equipment is perfect, and the rescue team is hardly injured
	4	The equipment is relatively complete, and the rescue team has safety guarantee
	3	The equipment still needs to be supplemented and perfected, but the rescue team can perform basic emergency rescue tasks relatively safely
	2	The equipment is not perfect, and there are certain difficulties for rescue team members to perform emergency rescue tasks safely
	1	The equipment is imperfect, and the rescue team cannot get effective safety protection

*The ability of emergency rescue equipment to control and reduce the impact of an accident D19*	5	It has obvious effects on controlling the source of danger and reducing the consequences of accidents
	4	It has a relatively obvious effect on controlling the source of danger and reducing the consequences of accidents
	3	It has a certain obvious effect on controlling the source of danger and reducing the consequences of accidents
	2	Has a small effect on controlling the source of danger and reducing the consequences of accidents
	1	No effect on controlling hazards and reducing the consequences of accidents

*Protective capabilities of personal protective equipment D20*	5	Strong protection
	4	Relatively strong protection
	3	General protection
	2	Relatively weak protection
	1	Weak protection

*Completeness of emergency materials D21*	5	A complete range of emergency supplies and can perform all emergency activities
	4	A relatively complete range of emergency supplies and can perform most emergency activities
	3	A complete range of emergency supplies and can perform basic emergency activities, but the types of emergency supplies still need to be optimized
	2	The types of emergency supplies are not very complete, and there are difficulties in implementing emergency activities
	1	The types of emergency supplies are not complete, and it is difficult to implement emergency activities

*Replenishment of emergency materials D22*	5	Emergency supplies can be supplemented in time
	4	Emergency supplies can be replenished in a short time
	3	Emergency supplies cannot be supplemented in the short term, but subsequent supplementation will not affect the normal emergency response
	2	Emergency supplies will take a long time to be replenished
	1	Emergency supplies cannot be replenished

*Emergency funding support in Shanghai D23*	5	Give strong support and provide sufficient capital guarantee
	4	Give relatively strong support and provide more financial protection
	3	Give a certain level of support
	2	Give very little support
	1	Almost no support

*Territory emergency funding support D24*	5	Give strong support and provide sufficient capital guarantee
	4	Give relatively strong support and provide more financial protection
	3	Give a certain level of support
	2	Give very little support
	1	Almost no support

*Emergency rescue expertise training D25*	5	There is continuous professional knowledge training, and the effect is very good
	4	There is continuous professional knowledge training, and the effect is relatively good
	3	There is continuous professional knowledge training, but the effect is average
	2	There is continuous professional knowledge training, but the effect is very small
	1	There is no continuous professional knowledge training

*Emergency rescue professional skills training D26*	5	There is continuous professional skills training, and the effect is very good
	4	There is continuous professional skills training, and the effect is relatively good
	3	There is continuous professional skills training, but the effect is average
	2	There is continuous professional skills training, but the effect is very small
	1	There is no continuous professional skill training

*Workload of emergency drill D27*	5	Fully meet the requirements
	4	More satisfying requirements
	3	Generally meet the requirements
	2	Rarely meet the requirements
	1	Does not meet the requirements

*Effect of emergency drill D28*	5	The effect is obvious
	4	The effect is relatively obvious
	3	General effect
	2	Has little effect
	1	Has no effect

**Table 4:** Scoring table of professional rescue team for hazardous chemical accidents.

	Team 1	Team 2	Team 3	Team 4	Team 5	Team 6	Team 7	Team 8	Team 9	Team 10	Team 11
D1	1	3	4	4	5	4	5	3	5	5	4
D2	5	4	5	4	5	4	5	3	5	5	3
D3	4	3	5	4	5	4	5	3	5	5	4
D4	3	4	4	3	4	4	4	3	3	3	3
D5	3	5	5	5	5	4	5	3	3	3	4
D6	3	4	4	4	4	5	5	3	3	3	3
D7	5	3	4	2	4	5	4	1	3	1	4
D8	2	4	4	2	4	4	4	3	3	3	3
D9	2	3	5	2	3	4	3	3	3	3	3
D10	4	4	4	3	4	4	4	3	3	3	4
D11	1	1	5	5	5	1	5	1	5	5	5
D12	3	4	5	3	5	3	5	3	3	3	4
D13	3	3	5	4	5	4	5	3	3	3	4
D14	3	3	4	4	4	5	5	3	3	3	4
D15	3	4	5	4	5	3	4	1	3	1	4
D16	3	2	4	3	4	4	4	3	3	3	4
D17	3	3	5	3	4	3	4	3	3	3	5
D18	3	2	4	4	5	3	4	3	3	3	4
D19	3	2	4	4	5	3	5	3	5	3	4
D20	3	3	5	4	4	4	5	3	5	3	4
D21	3	3	5	3	4	3	4	3	5	3	5
D22	2	3	5	4	5	5	5	5	5	3	5
D23	2	1	3	3	1	3	1	3	3	3	3
D24	2	2	3	3	2	3	1	3	3	1	3
D25	3	3	5	1	4	2	5	1	3	1	5
D26	3	3	5	2	4	1	5	1	3	3	5
D27	4	2	4	4	5	4	5	3	3	3	5
D28	3	2	4	4	4	4	5	3	3	3	4

**Table 5:** Eigenvalue of the correlation coefficient matrix, contribution rate, and cumulative contribution rate of principal components.

Principal components	Eigen value	Contribution rate (%)	Cumulative contribution rate (%)
*P*_1_	12.431	44.395	44.395
*P*_2_	4.242	15.150	59.544
*P*_3_	2.848	10.172	69.716
*P*_4_	2.382	8.505	78.222
*P*_5_	2.135	7.627	85.849
*P*_6_	1.413	5.046	90.894
*P*_7_	1.078	3.849	94.743
*P*_8_	0.713	2.546	97.288
*P*_9_	0.489	1.747	99.036
*P*_10_	0.270	0.964	100.000
*P*_11_	9.714 × 10^–16^	3.469 × 10^–15^	100.000
*P*_12_	5.642 × 10^–16^	2.015 × 10^–15^	100.000
*P*_13_	4.890 × 10^–16^	1.746 × 10^–15^	100.000
*P*_14_	3.687 × 10^–16^	1.317 × 10^–15^	100.000
*P*_15_	3.357 × 10^–16^	1.199 × 10^–15^	100.000
*P*_16_	2.120 × 10^–16^	7.570 × 10^–16^	100.000
*P*_17_	1.229 × 10^–16^	4.390 × 10^–16^	100.000
*P*_18_	5.703 × 10^–17^	2.037 × 10^–16^	100.000
*P*_19_	−4.248 × 10^–17^	−1.517 × 10^–16^	100.000
*P*_20_	−1.253 × 10^–16^	−4.475 × 10^–16^	100.000
*P*_21_	−1.701 × 10^–16^	−6.075 × 10^–16^	100.000
*P*_22_	−2.841 × 10^–16^	−1.015 × 10^–15^	100.000
*P*_23_	−3.203 × 10^–16^	−1.144 × 10^–15^	100.000
*P*_24_	−5.065 × 10^–16^	−1.809 × 10^–15^	100.000
*P*_25_	−5.375 × 10^–16^	−1.920 × 10^–15^	100.000
*P*_26_	−8.421 × 10^–16^	−3.008 × 10^–15^	100.000
*P*_27_	−8.752 × 10^–16^	−3.126 × 10^–15^	100.000
*P*_28_	−2.195 × 10^–15^	−7.839 × 10^–15^	100.000

**Table 6:** Load matrix of principal components.

	*P*_1_	*P*_2_	*P*_3_	*P*_4_	*P*_5_	*P*_6_	*P*_7_
D1	0.522	0.444	−0.044	−0.579	0.267	−0.136	−0.078
D2	0.312	0.001	−0.688	−0.286	0.078	0.466	0.249
D3	0.621	0.444	−0.437	−0.248	0.075	0.368	0.021
D4	0.615	−0.680	0.086	−0.296	0.219	0.034	0.039
D5	0.661	−0.418	0.029	−0.152	−0.041	−0.491	0.236
D6	0.561	−0.513	0.260	−0.514	−0.175	0.048	0.158
D7	0.545	−0.424	0.059	0.381	−0.226	0.491	0.218
D8	0.572	−0.469	0.149	−0.311	0.503	0.023	−0.198
D9	0.478	−0.119	0.408	−0.072	0.628	0.286	−0.161
D10	0.570	−0.686	−0.008	0.373	−0.078	0.180	−0.098
D11	0.592	0.646	−0.331	−0.118	0.073	−0.209	0.023
D12	0.849	−0.279	−0.215	0.069	0.190	−0.253	−0.140
D13	0.944	−0.058	0.093	−0.112	−0.158	−0.107	−0.034
D14	0.742	−0.151	0.393	−0.277	−0.354	0.112	−0.038
D15	0.764	−0.274	−0.068	0.250	−0.026	−0.228	0.430
D16	0.812	0.207	0.289	0.035	−0.221	0.273	−0.279
D17	0.786	0.110	0.061	0.462	0.170	−0.140	−0.272
D18	0.761	0.347	−0.039	0.027	−0.361	−0.214	−0.073
D19	0.711	0.541	−0.173	−0.087	−0.115	−0.023	0.220
D20	0.785	0.323	0.095	−0.093	0.179	0.148	0.374
D21	0.655	0.431	−0.023	0.404	0.424	0.041	0.117
D22	0.609	0.337	0.567	−0.155	0.199	−0.124	−0.028
D23	−0.294	0.652	0.519	0.105	0.115	0.239	−0.025
D24	−0.054	0.255	0.756	0.374	0.119	−0.063	0.414
D25	0.809	−0.158	−0.183	0.458	0.175	0.039	−0.028
D26	0.727	0.052	−0.476	0.366	0.181	−0.096	−0.151
D27	0.758	0.171	0.042	0.179	−0.566	0.056	−0.174
D28	0.802	0.218	0.194	−0.161	−0.437	0.057	−0.110

**Table 7:** Coefficients of linear expressions of principal components.

	*P*_1_	*P*_2_	*P*_3_	*P*_4_	*P*_5_
D1	0.148	0.215	−0.026	−0.375	0.182
D2	0.088	0.001	−0.408	−0.185	0.053
D3	0.176	0.216	−0.259	−0.161	0.051
D4	0.175	−0.330	0.051	−0.192	0.150
D5	0.187	−0.203	0.017	−0.099	−0.028
D6	0.159	−0.249	0.154	−0.333	−0.120
D7	0.155	−0.206	0.035	0.247	−0.154
D8	0.162	−0.228	0.088	−0.201	0.344
D9	0.136	−0.058	0.242	−0.046	0.430
D10	0.162	−0.333	−0.005	0.242	−0.054
D11	0.168	0.314	−0.196	−0.076	0.050
D12	0.241	−0.135	−0.128	0.045	0.130
D13	0.268	−0.028	0.055	−0.073	−0.108
D14	0.211	−0.073	0.233	−0.180	−0.242
D15	0.217	−0.133	−0.041	0.162	−0.018
D16	0.230	0.100	0.171	0.023	−0.151
D17	0.223	0.053	0.036	0.299	0.116
D18	0.216	0.169	−0.023	0.017	−0.247
D19	0.202	0.263	−0.103	−0.056	−0.079
D20	0.223	0.157	0.056	−0.060	0.123
D21	0.186	0.209	−0.013	0.262	0.290
D22	0.173	0.163	0.336	−0.100	0.136
D23	−0.083	0.316	0.308	0.068	0.079
D24	−0.015	0.124	0.448	0.242	0.082
D25	0.229	−0.077	−0.109	0.297	0.119
D26	0.206	0.025	−0.282	0.237	0.124
D27	0.215	0.083	0.025	0.116	−0.388
D28	0.228	0.106	0.115	−0.105	−0.299

**Table 8:** Weight of principal components.

	*P*_1_	*P*_2_	*P*_3_	*P*_4_	*P*_5_
Weight (%)	51.71	17.65	11.85	9.91	8.88

**Table 9:** Scores of 11 professional rescue teams represented by 5 principal components.

	Team 1	Team 2	Team 3	Team 4	Team 5	Team 6	Team 7	Team 8	Team 9	Team 10	Team 11
*P*_1_	14.692	14.99	22.363	16.775	21.986	17.475	22.824	13.054	17.386	14.646	20.322
*P*_2_	−0.436	−2.535	1.908	3.116	1.631	−0.25	1.36	1.863	4.459	3.459	3.038
*P*_3_	0.368	1.219	1.658	2.222	0.295	5.034	0.078	4.189	1.105	−0.113	2.022
*P*_4_	1.305	−0.836	0.252	−2.469	−1.367	−2.422	−1.932	−1.889	−1.457	−3.445	1.742
*P*_5_	−0.415	2.966	3.591	−0.181	1.187	0.791	1.075	1.743	3.325	2.327	1.713
Comprehensive score	7.657	7.631	12.441	9.225	11.662	9.418	11.956	7.541	10.058	8.035	11.607

**Table 10:** Normalized statistics of 11 professional rescue teams for hazardous chemical accidents.

	Team 1	Team 2	Team 3	Team 4	Team 5	Team 6	Team 7	Team 8	Team 9	Team 10	Team 11
*P*_1_	−0.665	−0.604	0.906	−0.238	0.828	−0.095	1.000	−1.000	−0.113	−0.674	0.488
*P*_2_	−0.400	−1.000	0.271	0.616	0.191	−0.347	0.114	0.258	1.000	0.714	0.594
*P*_3_	−0.813	−0.482	−0.312	−0.093	−0.841	1.000	−0.926	0.672	−0.527	−1.000	−0.170
*P*_4_	0.832	0.006	0.425	−0.624	−0.199	−0.606	−0.417	−0.400	−0.233	−1.000	1.000
*P*_5_	−1.000	0.688	1.000	−0.883	−0.200	−0.398	−0.256	0.077	0.867	0.369	0.062
Comprehensive score	−0.953	−0.963	1.000	−0.313	0.682	−0.234	0.802	−1.000	0.027	−0.798	0.660

**Table 11:** Results of five training functions on training samples.

Training function	Algorithm	Number of iterations	Convergence accuracy
trainlm	LevenbergMarquardt method	5	4.4339 × 10^–24^
traingd	Gradient descent method	2000	0.0022997
traingdm	Gradient descent method with momentum factor	2000	0.0021524
traingda	Gradient descent method with adaptive learning rate	2000	5.854 × 10^–08^
traingdx	Gradient descent method with adaptive learning rate and momentum factor	313	8.3968 × 10^–10^

**Table 12:** Training results of different numbers of neurons in the hidden layer.

Number of hidden neurons	Number of iterations	Convergence accuracy
4	268	9.7354 × 10^–6^
5	337	9.4521 × 10^–6^
6	187	8.8167 × 10^–6^
7	193	9.9709 × 10^–6^
8	144	9.7153 × 10^–6^
9	125	8.6169 × 10^–6^
10	242	9.7497 × 10^–6^
11	240	8.8618 × 10^–6^
12	138	8.7017 × 10^–6^
13	120	5.8329 × 10^–6^

**Table 13:** Relative error between training result and theoretical comprehensive score.

	Team 1	Team 2	Team 3	Team 4	Team 5	Team 6	Team 7	Team 8
Comprehensive score	7.657	7.631	12.441	9.225	11.662	9.418	11.956	7.541
Predicted result	7.655	7.632	12.438	9.225	11.679	9.415	11.943	7.543
Relative error (%)	0.026	0.013	0.024	0.000	0.146	0.032	0.109	0.027

**Table 14:** Relative error between prediction result and theoretical comprehensive score.

	Team 9	Team 10	Team 11
Comprehensive score	10.058	8.035	11.607
Predicted result	10.661	7.5	11.699
Relative error (%)	5.995	6.658	0.793

## Data Availability

All the data used to support this study have been included within the article.

## References

[1] Zhao B. (2016). Facts and lessons related to the explosion accident in Tianjin Port, China. *Natural Hazards*.

[2] Wang X. Z., Sugumaran V., Zhang H., Xu Z. (2018). A capability assessment model for emergency management organizations. *Information Systems Frontiers*.

[3] Lin L. Comprehensive evaluation study for urban emergency rescue capability.

[4] Yang F. F., Gai K., Cao F. (2019). Analysis of key elements of emergency response ability of hazardous chemicals based on AHP method. *E3S Web of Conferences*.

[5] Yu Z. F., Guan J. L. (2016). Fire and rescue combat technical training system construction for dangerous chemicals. *Procedia Engineering*.

[6] Zhu R. C., Li X., Hu X. F., Hu D. S. (2020). Risk analysis of chemical plant explosion accidents based on Bayesian network. *Sustainability*.

[7] He N., Jin L. Z., Wu Z. Z., Xu B. An evaluation methodology for emergency response capability in chemical accidents.

[8] Yuan Y., Zhou X., Man J. (2019). The safety evaluation of management in chemical enterprise with generalized regression neural network. *IOP Conference Series: Earth and Environmental Science*.

[9] Yang Q. Study on evaluation of chemical industry safety production based on artificial neural network.

[10] Gao Y., Cao Y., Jiang Z. (2019). Investment forecast of power network infrastructure project based on BP neural network. *IOP Conference Series: Earth and Environmental Science*.

[11] Xia L. M., Zhao C. X. The application of PCA-fuzzy probability analysis on risk evaluation of construction schedule of highway.

[12] Zhang H. J., Li Y. L., Zhang H. L. (2019). Risk early warning safety model for sports events based on back propagation neural network machine learning. *Safety Science*.

[13] Yang F. (2018). Construction of evaluation model of university student education evaluation system and its MATLAB simulation based on BP neural network algorithm. *Educational Sciences-Theory & Practice*.

[14] Yang C. W., Li Z. H., Guo X. Y., Yu W. Y., Jin J., Zhu L. (2019). Application of BP neural network model in risk evaluation of railway construction. *Complexity*.

[15] Deng X. H., Xu T., Wang R. (2018). Risk evaluation model of highway tunnel portal construction based on BP fuzzy neural network. *Computational Intelligence and Neuroscience*.

